# The use of linked data to develop and validate measures of variation in mortality and readmissions for public reporting purposes in New South Wales, Australia

**DOI:** 10.23889/ijpds.v1i1.311

**Published:** 2017-04-19

**Authors:** Kim Sutherland, Christopher Papadopoulos, Sadaf Marashi-Pour, Huei-Yang Chen, Jean-Fr'ed'eric L\&apos;evesque

**Affiliations:** Bureau of Health Information; Centre for Primary Health Care and Equity, University of New South Wales

## Objectives

To investigate variation across 78 New South Wales public hospitals, in mortality in the 30 days following admission and in returns to acute care (readmissions) in the 30 days following discharge for acute myocardial infarction, ischaemic stroke, heart failure, pneumonia, hip fracture surgery.

## Approach

Linked data were used to (1) construct an analytic unit - an index period of care that comprised concatenated acute, contiguous hospitalisations with the principal diagnosis of interest; (2) to capture outcomes both within the index hospital and following discharge, wherever they occurred; (3) to enhance risk adjustment with one year look back for relevant comorbidities; (4) to assess fair attribution of outcomes. A risk-standardised mortality ratio (RSMR) and a risk standardised readmission ratio (RSRR) were calculated as the ratio of the observed to the expected number events at a given hospital, by developing and validating condition specific system-level prediction models. Funnel plots identified outliers. For the RSRR, the competing risk of death was considered. 

## Results

For both outcome indicators, sensitivity was enhanced by the use of linked data (33%-100% more deaths; 23%-32% more returns to acute care or readmissions). For mortality, RSMRs that only capture deaths in hospital, as opposed to deaths within 30 days of admission, were shown to be biased and change the outlier status of about 20% of hospitals. Including socioeconomic status in risk adjustment models altered the outlier status of about 10% of hospitals on the cusp of statistical significance but did not significantly alter the RSMRs. For returns to acute care, sensitivity analyses that included socioeconomic status in the models found there was no significant improvement in discriminatory power. For example, in the case of ischaemic stroke, the c-statistic for the model without inclusion of SES was 0.593 (0.578-0.610); inclusion of SES resulted in a c-statistic of 0.600 (0.583-0.616). There were some changes in hospital-level results but there was no clear evidence of a systematic effect on results.

## Conclusion

The risk-standardised ratio method, based on linked data, compares a hospital's results given its case mix with an average New South Wales hospital with the same case mix. Ratio-based indicators have been reported publicly and have proven to be a valuable screening tool to identify hospitals where further investigation may be required locally.

